# Time to Surgery Following Short-Course Radiotherapy in Rectal Cancer and its Impact on Postoperative Outcomes. A Population-Based Study Across the English National Health Service, 2009–2014

**DOI:** 10.1016/j.clon.2019.08.008

**Published:** 2020-02

**Authors:** B.A. Levick, A.J. Gilbert, K.L. Spencer, A. Downing, J.C. Taylor, P.J. Finan, D.J. Sebag-Montefiore, E.J.A. Morris

**Affiliations:** ∗ Leeds Institute for Data Analytics, University of Leeds, Leeds, UK; † Leeds Institute of Medical Research at St James's, School of Medicine, University of Leeds, Leeds, UK; ‡ Leeds Cancer Centre, St James' Hospital, Leeds, UK

**Keywords:** Cancer, interval, neoadjuvant, outcomes, rectal, short-course radiotherapy

## Abstract

**Aims:**

Preoperative short-course radiotherapy (SCRT) is an important treatment option for rectal cancer. The length of time between completing SCRT and surgery may influence postoperative outcomes, but the evidence available to determine the optimal interval is limited and often conflicting.

**Materials and methods:**

Information was extracted from a colorectal cancer data repository (CORECT-R) on all surgically treated rectal cancer patients who received SCRT in the English National Health Service between April 2009 and December 2014. The time from radiotherapy to surgery was described across the population. Thirty-day postoperative mortality, returns to theatre, length of stay and 1-year survival were investigated in relation to the interval between radiotherapy and surgery.

**Results:**

Within the cohort of 3469 patients, the time to surgery was 0–7 days for 76% of patients, 8–14 days for 19% of patients and 15–27 days for 5% of patients. There was a clear variation in relation to different patient characteristics. There was, however, no evidence of differences in postoperative outcomes in relation to interval length.

**Conclusions:**

This study suggests that the time interval between SCRT and surgery does not influence postoperative outcomes up to a year after surgery. The study provides population-level, real-world evidence to complement that from clinical trials.

## Highlights

• The optimal interval between SCRT and rectal surgery is not known.
• SCRT and surgery intervals vary substantially across the English NHS.
• There are no differences in surgical outcomes with intervals of up to 27 days after SCRT.

## Introduction

Neoadjuvant short-course radiotherapy (SCRT) is an effective treatment for rectal cancer. Randomised trials have shown that although it reduces the risk of locally recurrent disease, it does not influence survival [1], [2], [3], [4]. Furthermore, it may be associated with an increased risk of treatment-related morbidity [5]. Therefore, in routine practice, the potential benefits of the treatment must be balanced against its risks. In the UK, patients who have tumours that are neither low risk (T1-3aN0) nor high risk (circumferential resection margin (CRM) threatened) are recommended to be considered for SCRT, usually delivered as 25 Gy in five fractions with surgery undertaken immediately after the completion of radiotherapy [6], [7], [8]. The evidence base as to how long the ‘immediate’ time period should be is conflicting. Consequently, recommendations regarding the timing of surgery after the completion of radiotherapy vary widely internationally.

Within Europe, individual guidelines also vary, but generally state that surgery should be undertaken within 2–4 working days [2], [4], [7], [8], [9], [10], [11], [12] (Figure 1A). This recommendation arose from a subset analysis of the Dutch total mesorectal excision (TME) trial, which reported an increase in postoperative mortality in people over 75 years of age when the interval was 4–7 days compared with 0–3 days [10]. Despite an accompanying population-based study being unable to replicate the finding, these data have been used to support a policy of rapid surgery after SCRT.

**Fig 1:**
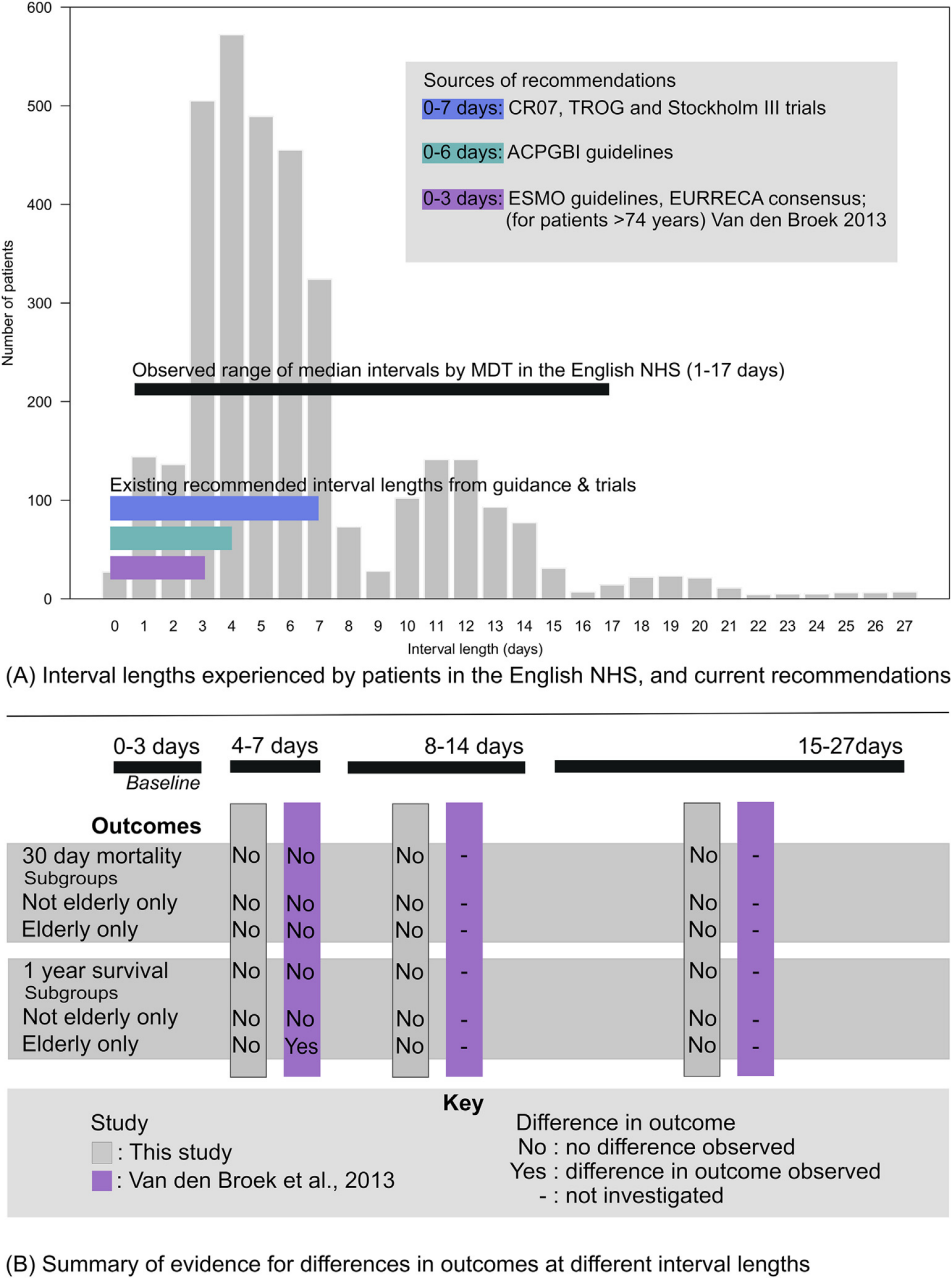
(A) Distribution of the length of the interval before receiving surgery in short-course radiotherapy (SCRT) patients across the English National Health Service and current recommendations for interval length; (B) summary of current evidence of differences in outcomes with different interval lengths.

The recent Stockholm III study randomised patients to three arms: SCRT (5 × 5 Gy) with surgery within 0–7 days, SCRT (5 × 5 Gy) with surgery within 28–56 days or long-course radiotherapy (25 × 2 Gy) and surgery within 28–56 days. It found that there were similar oncological results and postoperative mortality across all these groups, but postoperative complications were significantly reduced in the SCRT group with a longer interval (28–56 days) before surgery. They concluded that delaying surgery may, therefore, be a useful approach, with longer intervals being advantageous [13].

In England, little is known about the intervals adopted between SCRT and surgery or their impact on patient outcomes. Patterns of use and outcomes of both SCRT and surgery can, however, be determined from linked, routinely collected, population-based National Health Service (NHS) datasets [14]. Therefore, the aim of this study was to use these population-based data to examine the variation in SCRT to surgery intervals across the English NHS and to relate this to postoperative outcomes up to a year after surgery. It also aimed to determine if the findings of the Dutch TME trial [2], [10] were replicated in a population-based ‘real-world’ dataset.

## Materials and Methods

Data were obtained from the UK Colorectal Cancer Intelligence Hub's colorectal cancer data repository (CORECT-R), within which the National Cancer Registry and Analysis Services data are linked to many other datasets, including Hospital Episode Statistics and the National Radiotherapy Dataset.

Information was extracted on all individuals in the English NHS with a primary diagnosis of rectal cancer (ICD-10 C20) [15] made between 1 April 2009 and 31 December 2014, who received SCRT followed by a major resection. Patients who had records of either five (25 Gy in five fractions) or four (20 Gy in four fractions, delivered as an equivalent regimen at one centre) attendances for radiotherapy and subsequently underwent surgery in ≤27 days from their last attendance for radiotherapy were defined as receiving SCRT.

Those who had an interval >27 days were excluded as it was assumed that most of these individuals were probably receiving SCRT with an intended delay. This treatment is increasingly used as an alternative to long-course neoadjuvant chemoradiotherapy in individuals with more advanced tumours, where the risk of involved surgical margins is high, but concern exists about the patient's ability to tolerate chemoradiotherapy (due to frailty or comorbidity). In this context, the delay between radiotherapy and surgery is planned to allow the tumour to be downstaged before resection. Unfortunately, this precluded direct comparison of the results of these observational data with those of two arms of the Stockholm III study (SCRT with delay and long-course radiotherapy) as the inclusion criteria of the trial limited their population to those who were eligible for SCRT with more immediate surgery.

To enable an investigation of the impact of interval length between SCRT and surgery, the population was split into a number of groups. First, based on inspection of the time between SCRT and surgery across this cohort (Figure 1A), as well as clinical advice on typical working practices in English hospitals, individuals were grouped into categories of those with intervals of 0–7, 8–14 or 15–27 days. In addition, in order to make comparisons with the randomised trials that investigated interval length and mortality [2], [10], individuals were also subdivided into 0–3, 4–7, 8–14 and 15–27 day categories.

Information on variation in interval length in relation to the characteristics of the population and other relevant features were extracted from the linked datasets. These included comorbidity, which was scored using the Charlson index [16], and categorised as a score of 0, 1, 2 or ≥3. Operations were classified from OPCS4 codes using an algorithm defined previously [17] and consisted primarily of anterior resection, abdominoperineal excision or Hartmann's procedure. Tumour stage at diagnosis was taken from registry data. The patient's socioeconomic deprivation was categorised into groups using quintiles of the income domain of the English Index of Multiple Deprivation, derived from the patient's postcode at the time of diagnosis [18]. The patient's age at diagnosis was categorised as ≤61, 61–70, 71–80 or ≥81 years. Again, to enable comparisons with the previous study, a second grouping of ≥75 and < 75 years was created.

Postoperative outcomes included 30-day mortality, 1-year postoperative survival, rates of return to theatre [19] and length of stay.

The interval lengths experienced by key groups in the population were described. The median time interval and interquartile range (IQR) was calculated for each of the multidisciplinary teams managing rectal cancer in the English NHS. The rates of each postoperative outcome were calculated by interval length categories. Small cell counts in tables were suppressed in order to reduce the risk of disclosure.

The relationship between interval length and 1-year survival was assessed using a Cox proportional hazards model. Associations between interval length and length of stay were tested using a Poisson regression model. Associations between interval length and binomial outcomes (30-day mortality and returns to theatre) were tested using logistic regression models. In each case, the analysis was carried out as both univariable and multivariable models adjusted for gender, Charlson comorbidity score and Index of Multiple Deprivation quintile, stage of tumour at diagnosis and age (excluding independent models in those ≥75 years and <75 years).

The analysis was conducted using the R statistical computing environment [20] using the ‘survival’ [21], [22], ‘readstata13’ [23] and ‘xlsx’ [24] packages and STATA IC 15 [25].

## Results

In total, 3469 individuals received SCRT followed by surgery within ≤27 days across the whole study period. This represented 11% of all patients diagnosed with rectal cancer. Most (76.4%) underwent surgery within 7 days of finishing radiotherapy, 18.9% experienced an interval of 8–14 days and 4.7% an interval of 15–27 days (Table 1). Longer intervals (15–27 days) were more common among patients over the age of 80 years, those with greater comorbidity and in those where the tumour stage was not recorded.

**Table 1:** Characteristics of the population in relation to the interval between radiotherapy and surgery

Patient characteristics and procedures		Length of interval between finishing short-course radiotherapy and surgery						Total
		0–7 days		8–14 days		15–27 days		
		*n*	%	*n*	%	*n*	%	
Total number of patients experiencing each length of interval		2652	76.4	656	18.9	162	4.7	3469
Age	<61 years	679	76.9	174	19.7	30	3.4	883
	61–70 years	960	78.1	222	18.1	48	3.9	1230
	71–80	816	75.4	202	18.7	64	5.9	1082
	>80 years	197	71.9	57	20.8	20	7.3	274
Gender	Male	1793	76.0	451	19.1	114	4.8	2358
	Female	859	77.3	204	18.4	48	4.3	1111
Charlson comorbidity index	0	2198	76.8	547	19.1	118	4.1	2863
	1	345	75.8	81	17.8	29	6.4	455
	2+∗	109	72.2	27	17.9	15	9.9	151
Index of Multiple Deprivation quintile	1 – least deprived	559	76.2	139	18.9	36	4.9	734
	2	640	76.9	148	17.8	44	5.3	832
	3	577	78.6	127	17.3	30	4.1	734
	4	480	76.1	127	20.1	24	3.8	631
	5 – most deprived	396	73.6	114	21.2	28	5.2	538
Stage	I	527	73.5	158	22.0	32	4.5	717
	II	644	77.3	152	18.3	37	4.4	833
	III and IV∗	1209	78.8	259	16.9	67	4.4	1535
	Unknown	272	70.8	86	22.4	26	6.8	384
Procedure	Anterior resection	723	71.7	226	22.4	59	5.9	1008
	Abdominoperineal excision	1659	79.0	360	17.1	82	3.9	2101
	Hartmann's and other∗	270	75.0	69	19.2	21	5.8	360

∗ Aggregated to suppress small numbers.

The distribution of intervals between SCRT and surgery is shown in Figure 1A. The median interval length across individual multidisciplinary teams ranged from 1 day [IQR 0.5 days (1–1.5)] to 17 days [IQR 13 days (10–23)].

Although small differences in 30-day and 1-year postoperative mortality, rates of return to theatre and length of stay were observed between the interval length groups, none of these differences was statistically significant (*P* > 0.1) (Table 2; Figure 2; Supplementary Figure S1, Tables S1, S2). This was the case even when the analyses were stratified in the same age groups as those used in the influential subset analysis of the Dutch TME trial (Supplementary Tables S3–S5, Figure S2). A comparison of the interval lengths experienced in this English cohort with current recommendations in Europe and the results of the relevant trials are summarised in Figure 1B.

**Table 2:** Mortality (30 day and 1 year) and returns to surgery after major resection by interval length group

Length of interval (days)	30-day postoperative mortality		1-year postoperative mortality		Return to theatre within 28 days		Total
	*n*	%	*n*	%	*n*	%	
0–7	57	2.2	165	6.2	310	11.7	2652
8–14	11	1.7	47	7.2	63	9.6	656
15–27∗	<7	<4%	12	7.3	19	11.7	162

∗ Small numbers are suppressed.

**Fig 2:**
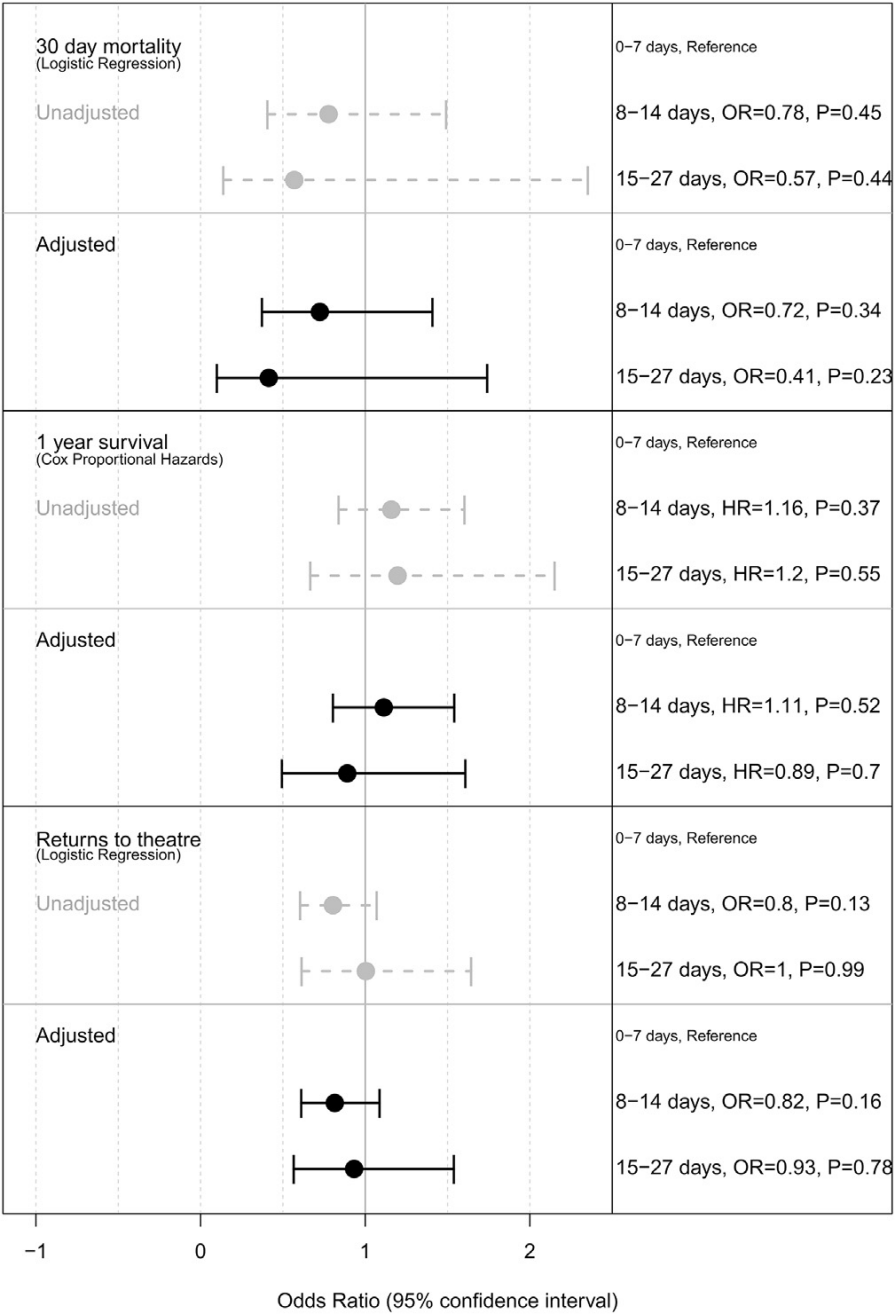
Association of interval length with 30-day mortality, 1-year survival and returns to theatre in short-course radiotherapy (SCRT) patients. Multivariate models (black solid lines) adjusted for age, gender, stage, Charlson index and Index of Multiple Deprivation category.

## Discussion

This study, carried out in the English NHS, is not able to provide any evidence of worse postoperative outcomes with a time to surgery of 4–7 days after SCRT compared with 0–3 days, irrespective of age. Furthermore, this study found no evidence of any other effect on outcomes when the interval to surgery was extended up to 27 days from the completion of SCRT. This shows the limitations of the Van den Broek *et al.* [10] subset analyses as well as the importance of observing the implementation and confirmation of trial findings in a real-world setting. As this is the largest population-based study to be undertaken investigating the effect of variations in SCRT to surgery intervals, these data provide important real-world evidence to help inform future guidance and so optimise the use of SCRT in the management of rectal cancer. Guidelines recommending intervals as short as 3–4 working days based on the Van den Broek *et al.* [10] study may, therefore, require revision.

This study also found that in the English NHS, although most patients operated on within 27 days of the completion of radiotherapy were operated on within 7 days (76.4%), there was substantial variation in the timing between SCRT and surgery. To an extent, this is not surprising given the variation in guidelines and recommendations for interval lengths [2], [4], [7], [8], [9], [10], [11], [12] (Figure 1A). Previous studies have also described variation in the radiotherapy regimens used to treat rectal cancer in the English NHS [14]. Although there is a relevant National Institute for Health and Care Excellence guideline in place [6], this persistent variation indicates a need for greater consensus on radiotherapy use for rectal cancer in the English NHS.

In contrast to Van den Broek *et al.* [10], the Stockholm III trial provided evidence to support longer delays of 28–56 days to surgery; finding reduced postoperative complications, albeit with a slightly increased risk of radiotherapy-related toxicity requiring readmission [13]. Unfortunately, it was not possible to investigate the effects of these longer intervals in this cohort as the routine data available did not allow us to distinguish those who would have met the eligibility criteria of the Stockholm III trial. This is because patients whose tumours are threatening, or involving, the planned resection margins are prescribed SCRT with an intended delay as an alternative (often due to frailty, poor performance status or comorbidity) to long-course radiotherapy or, indeed, long-course chemoradiotherapy, which is also often used in this setting. Given that these two populations pose different surgical risks they are also likely to experience different outcomes and, as we could not distinguish between them in the population-based English rectal cancer data, direct comparisons were precluded. This is a limitation of the routine data available and further detail on the planned use of SCRT would be required before any such comparative analyses could be undertaken.

Defining the optimal interval remains a challenge. Most current SCRT trial protocols allow up to 7 days from the completion of SCRT to surgery. The advantages of the 7-day interval are that there is a limited time for downstaging of the tumour, which allows decisions regarding stage and risk stratification to be made on the basis of the pathological stage. Shorter intervals also allow for planned adjuvant therapy to start sooner, and so reduce the overall treatment time. In contrast, with longer intervals, a downstaging effect becomes more likely, and pretreatment restaging imaging may be required to guide postoperative treatment planning. Based on the findings of this study, if ‘immediate’ surgery is intended it seems reasonable to allow up to 7 days from the completion of SCRT. This would simplify postoperative decision-making, allowing plans to be made based on histopathological findings at surgery.

The Stockholm III trial was specifically designed to compare oncological outcomes between patients receiving SCRT with immediate surgery, with delayed surgery or long-course radiotherapy, and so provides the gold standard evidence base. As a consequence, SCRT with a 28–56-day interval is considered to be an acceptable alternative to SCRT with immediate surgery. As this study was not able to allocate the population to the trial arms used by the Stockholm III trial, its value in evaluating whether this recommendation is applicable in a real-world setting is limited.

This study has a number of other limitations arising from its use of linked routine administrative data from the English NHS. For example, as recurrence data are not routinely captured it was not possible to assess the effect of interval length on this outcome. Similarly, the analysis was limited by a lack of data on important aspects of radiotherapy-related toxicity, intra- or postoperative morbidity, such as infection, cardiovascular or respiratory events. Acute pelvic toxicity associated with radiotherapy and longer term functional outcomes, as reported by physicians or patients, are also not routinely collected. Efforts are underway, however, to increase the scope of the routine data available and so, in the future, they may support more detailed and informative observational analyses.

This is, however, the largest population-based observational study investigating the effect of the interval length between SCRT and surgery and so provides valuable data to inform optimal practice. We found no evidence of worsened mortality outcomes after intervals of 4–7, 8–14 or 15–27 days compared with intervals of 0–3 days. Other studies have shown the increasing impact of downstaging beyond 7 days. Therefore, the current guidelines and recommended intervals in trials support an interval length of up to 7 days. Interval lengths of between 28 and 56 days can also be used in appropriate populations. This study shows the importance of investigating the results of trials in a real-world setting and the dangers of using subset analysis of randomised trials to inform clinical policy.

## Conclusions

In conclusion, the study shows that time intervals of 4 days or more between SCRT and surgery are not associated with worse postoperative outcomes up to a year after surgery for rectal cancer patients.

## Conflict of Interest

The authors declare no conflicts of interest.

## Figures and Tables

**figs1:**
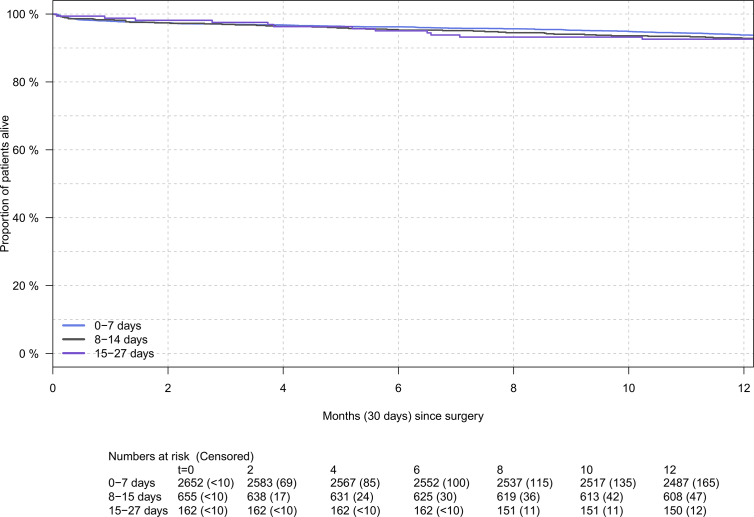


**figs2:**
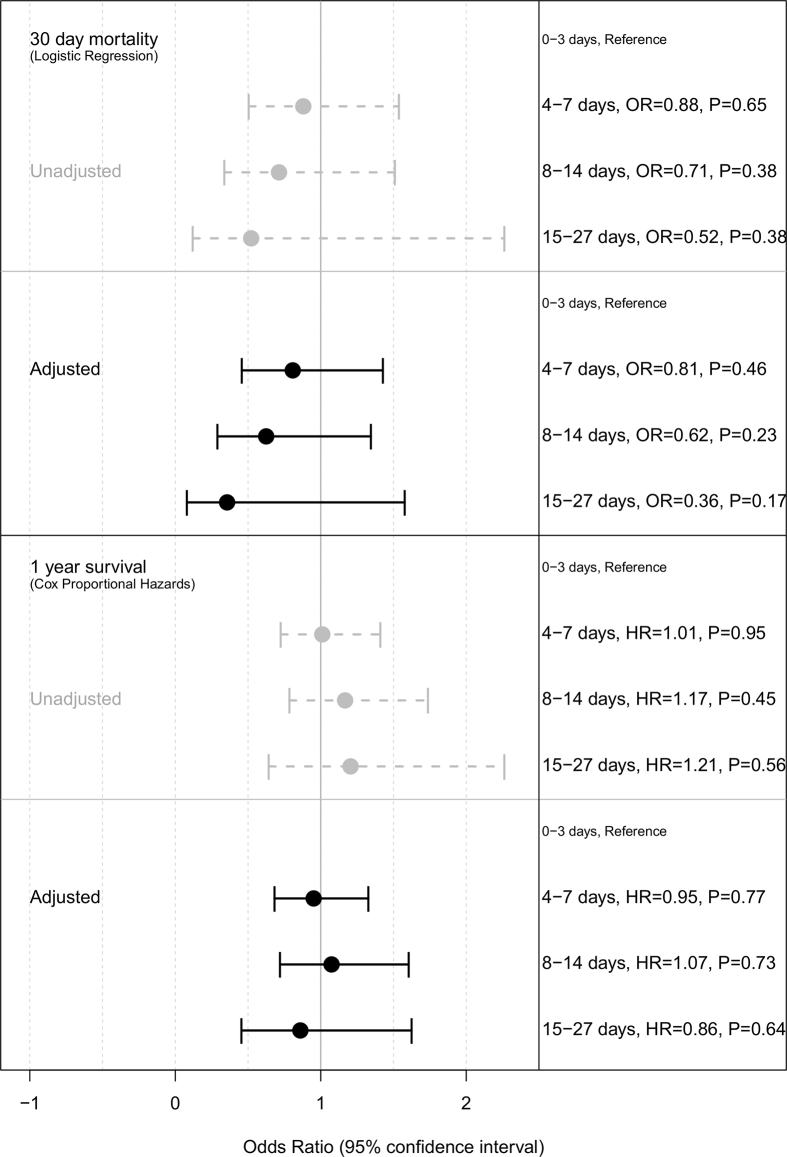
2
