# Supplementary material for: Time to Surgery Following Short-Course Radiotherapy in Rectal Cancer and its Impact on Postoperative Outcomes. A Population-Based Study Across the English National Health Service, 2009–2014
Source: Clin Oncol (R Coll Radiol). 2020 Feb;32(2):e46–52. doi: 10.1016/j.clon.2019.08.008 (PMC6966322; doi:10.1016/j.clon.2019.08.008)
Supplement: Multimedia component 1 [file mmc1.docx]

**Fig S1.** Unadjusted one-year survival by length of interval between finishing radiotherapy and surgery in patients receiving SCRT.

**Fig S2.** Association of interval length with 30-day mortality & one-year survival in SCRT patients, considering shorter interval lengths as per Van den Broek *et al.*, 2013. Multivariate models (in black solid lines) adjusted for age, sex, stage, Charlson Index and IMD category.
