# Supplementary material for: Time to Surgery Following Short-Course Radiotherapy in Rectal Cancer and its Impact on Postoperative Outcomes. A Population-Based Study Across the English National Health Service, 2009–2014
Source: Clin Oncol (R Coll Radiol). 2020 Feb;32(2):e46–52. doi: 10.1016/j.clon.2019.08.008 (PMC6966322; doi:10.1016/j.clon.2019.08.008)
Supplement: Multimedia component 2 [file mmc2.docx]

**Supplementary Table S1**

Associations between interval length and thirty day mortality, one year survival, and returning to theatre. Models are adjusted for patient age, stage, co-morbidity, sex and IMD.

|  | 30 day mortality - logistic regression | | | | | 1 year survival - Cox Proportional Hazards | | | | | Return to theatre - logistic regression | | | | |
| --- | --- | --- | --- | --- | --- | --- | --- | --- | --- | --- | --- | --- | --- | --- | --- |
|  | Unadjusted | | | | | Unadjusted | | | | | Unadjusted | | | | |
| Predictor |  | OR | CI - lower | CI - higher | P |  | HR | CI - lower | CI - higher | P |  | OR | CI - lower | CI - higher | P |
| Interval length | 0-7 days | *Reference* |  |  |  | 0-7 days | *Reference* |  |  |  | 0-7 days | *Reference* |  |  |  |
|  | 8-14 days | 0.78 | 0.41 | 1.49 | 0.45 | 8-14 days | 1.16 | 0.84 | 1.60 | 0.37 | 8-14 days | 0.80 | 0.60 | 1.07 | 0.13 |
|  | 15-27 days | 0.57 | 0.14 | 2.35 | 0.44 | 15-27 days | 1.20 | 0.67 | 2.15 | 0.55 | 15-27 days | 1.00 | 0.61 | 1.64 | 0.99 |
|  | *Baseline* | 0.02 | 0.02 | 0.03 | <0.001 |  |  |  |  |  | *Baseline* | 0.13 | 0.12 | 0.15 | <0.001 |
|  | Adjusted | | | | | Adjusted | | | | | Adjusted | | | | |
|  |  | OR | CI - lower | CI - higher | P |  | HR | CI - lower | CI - higher | P |  | OR | CI - lower | CI - higher | P |
| Interval length | 0-7 days | *Reference* |  |  |  | 0-7 days | *Reference* |  |  |  | 0-7 days | *Reference* |  |  |  |
|  | 8-14 days | 0.72 | 0.37 | 1.41 | 0.34 | 8-14 days | 1.11 | 0.80 | 1.54 | 0.52 | 8-14 days | 0.82 | 0.61 | 1.09 | 0.16 |
|  | 15-27 days | 0.41 | 0.10 | 1.74 | 0.23 | 15-27 days | 0.89 | 0.49 | 1.61 | 0.70 | 15-27 days | 0.93 | 0.57 | 1.54 | 0.78 |
|  | *Baseline* | 0.06 | 0.03 | 0.17 | <0.001 |  |  |  |  |  | *Baseline* | 0.09 | 0.05 | 0.15 | <0.001 |
