# Supplementary material for: Time to Surgery Following Short-Course Radiotherapy in Rectal Cancer and its Impact on Postoperative Outcomes. A Population-Based Study Across the English National Health Service, 2009–2014
Source: Clin Oncol (R Coll Radiol). 2020 Feb;32(2):e46–52. doi: 10.1016/j.clon.2019.08.008 (PMC6966322; doi:10.1016/j.clon.2019.08.008)
Supplement: Multimedia component 3 [file mmc3.docx]

**Supplementary Table S2**

Associations between interval length and additional postoperative outcomes. All models are logistic regression unless indicated. Adjusted models are adjusted for patient age, stage, co-morbidity, sex and IMD.

|  | Return to theatre due to stoma complications | | | | | | | |
| --- | --- | --- | --- | --- | --- | --- | --- | --- |
| Interval | Unadjusted |  |  |  | Adjusted |  |  |  |
| Length | OR | CI Low | CI High | P | OR | CI Low | CI High | P |
| Baseline | 0.18 | 0.13 | 0.24 | <0.001 | 0.06 | 0.01 | 0.29 | <0.001 |
| 0-7 days | *Reference* |  |  |  |  |  |  |  |
| 8-14 days | 1.18 | 0.58 | 2.43 | 0.65 | 1.26 | 0.59 | 2.69 | 0.55 |
| 15-27 days | 0.66 | 0.15 | 2.94 | 0.58 | 0.40 | 0.08 | 1.98 | 0.26 |
|  | Return to theatre for laparotomy procedures | | | | | | | |
|  | Unadjusted |  |  |  | Adjusted |  |  |  |
|  | OR | CI Low | CI High | P | OR | CI Low | CI High | P |
| Baseline | 1.90 | 1.50 | 2.40 | 0.00 | 1.72 | 0.60 | 4.91 | 0.31 |
| 0-7 days | *Reference* |  |  |  |  |  |  |  |
| 8-14 days | 1.32 | 0.73 | 2.39 | 0.36 | 1.42 | 0.75 | 2.70 | 0.29 |
| 15-27 days | 0.59 | 0.23 | 1.49 | 0.26 | 0.86 | 0.30 | 2.49 | 0.78 |
|  | Return to theatre for other procedures | | | | | | | |
|  | Unadjusted |  |  |  | Adjusted |  |  |  |
|  | OR | CI Low | CI High | P | OR | CI Low | CI High | P |
| Baseline | 0.07 | 0.04 | 0.11 | <0.001 | 0.38 | 0.08 | 1.91 | 0.24 |
| 0-7 days | *Reference* |  |  |  |  |  |  |  |
| 8-14 days | 0.98 | 0.32 | 2.98 | 0.98 | 0.81 | 0.26 | 2.59 | 0.73 |
| 15-27 days | 1.71 | 0.37 | 7.91 | 0.49 | 1.59 | 0.27 | 9.24 | 0.60 |
|  | Return to theatre due to wound complications | | | | | | | |
|  | Unadjusted |  |  |  | Adjusted |  |  |  |
|  | OR | CI Low | CI High | P | OR | CI Low | CI High | P |
| Baseline | 0.49 | 0.39 | 0.62 | <0.001 | 0.32 | 0.11 | 0.93 | 0.04 |
| 0-7 days | *Reference* |  |  |  |  |  |  |  |
| 8-14 days | 0.53 | 0.28 | 1.02 | 0.06 | 0.53 | 0.27 | 1.04 | 0.07 |
| 15-27 days | 1.48 | 0.58 | 3.80 | 0.41 | 1.24 | 0.46 | 3.39 | 0.67 |
|  | Length of stay (Poisson regression) | | | | | | | |
|  | Unadjusted |  |  |  | Adjusted |  |  |  |
|  | Coefficient | CI Low | CI High | P | Coefficient | CI Low | CI High | P |
| Baseline | 14.20 | 14.06 | 14.34 | <0.001 | 2.75 | 2.71 | 2.79 | <0.001 |
| 0-7 days | *Reference* |  |  |  |  |  |  |  |
| 8-14 days | 1.01 | 0.99 | 1.03 | 0.32 | 0.01 | -0.01 | 0.03 | 0.34 |
| 15-27 days | 1.08 | 1.04 | 1.13 | <0.001 | 0.00 | -0.04 | 0.04 | 0.87 |
