# Supplementary material for: Time to Surgery Following Short-Course Radiotherapy in Rectal Cancer and its Impact on Postoperative Outcomes. A Population-Based Study Across the English National Health Service, 2009–2014
Source: Clin Oncol (R Coll Radiol). 2020 Feb;32(2):e46–52. doi: 10.1016/j.clon.2019.08.008 (PMC6966322; doi:10.1016/j.clon.2019.08.008)
Supplement: Multimedia component 4 [file mmc4.docx]

**Supplementary Table S3**

Association between interval length and thirty day mortality and one-year survival, with additional interval length groups to align with analysis by Van den Broek *et al.*. Models are adjusted for patient age, stage, co-morbidity, sex and IMD.

|  | 30 day mortality - logistic regresssion | | | | | 1 year survival - Cox Proportional Hazards | | | | |
| --- | --- | --- | --- | --- | --- | --- | --- | --- | --- | --- |
|  | Unadjusted | | | | | Unadjusted | | | | |
| Predictor |  | OR | CI - lower | CI - higher | P |  | HR | CI - lower | CI - higher | P (z) (1 year) |
| Interval length | 0-3 days | *Reference* |  |  |  | 0-3 days | *Reference* |  |  |  |
|  | 4-6 days | 0.88 | 0.50 | 1.54 | 0.65 | 4-6 days | 1.01 | 0.72 | 1.41 | 0.95 |
|  | 7-14 days | 0.71 | 0.34 | 1.51 | 0.38 | 7-14 days | 1.17 | 0.78 | 1.74 | 0.45 |
|  | 15-27 days | 0.52 | 0.12 | 2.26 | 0.38 | 15-27 days | 1.21 | 0.64 | 2.26 | 0.56 |
|  | *Baseline* | 0.02 | 0.02 | 0.04 | <0.001 |  |  |  |  |  |
|  | Adjusted | | | | | Adjusted | | | | |
|  |  | OR | CI - lower | CI - higher | P |  | HR | CI - lower | CI - higher | P (z) (1 year) |
| Interval length | 0-3 days | *Reference* |  |  |  | 0-3 days | *Reference* |  |  |  |
|  | 4-6 days | 0.81 | 0.46 | 1.43 | 0.46 | 4-6 days | 0.95 | 0.68 | 1.33 | 0.77 |
|  | 7-14 days | 0.62 | 0.29 | 1.35 | 0.23 | 7-14 days | 1.07 | 0.72 | 1.60 | 0.73 |
|  | 15-27 days | 0.36 | 0.08 | 1.58 | 0.17 | 15-27 days | 0.86 | 0.46 | 1.62 | 0.64 |
