# Supplementary material for: Time to Surgery Following Short-Course Radiotherapy in Rectal Cancer and its Impact on Postoperative Outcomes. A Population-Based Study Across the English National Health Service, 2009–2014
Source: Clin Oncol (R Coll Radiol). 2020 Feb;32(2):e46–52. doi: 10.1016/j.clon.2019.08.008 (PMC6966322; doi:10.1016/j.clon.2019.08.008)
Supplement: Multimedia component 5 [file mmc5.docx]

**Supplementary Table S4**

Thirty day and one-year mortality in all patients, and split by not elderly/elderly, in interval length groups to align with analysis by Van den Broek *et al.*.

|  |  |  | Mortality at 30 days | | | | | Mortality at 1 year | | | | |
| --- | --- | --- | --- | --- | --- | --- | --- | --- | --- | --- | --- | --- |
|  |  |  | Alive | % | Dead | % | Total | Alive | % | Dead | % | Total |
| All patients | Interval length | 0-3 days | 793 | 97.66 | 19 | 2.34 | 812 | 762 | 93.84 | 50 | 6.16 | 812 |
|  |  | 4-7 days | 1802 | 97.93 | 38 | 2.07 | 1840 | 1725 | 93.75 | 115 | 6.25 | 1840 |
|  |  | 8-14 days | 644 | 98.32 | 11 | 1.68 | 655 | 608 | 92.82 | 47 | 7.18 | 655 |
|  |  | 15-27 days | 160 | 98.77 | <10 | <6 | 162 | 150 | 92.59 | 12 | 7.41 | 162 |
|  |  |  |  |  |  |  |  |  |  |  |  |  |
| Not elderly <75 years | Interval length | 0-3 days | 611 | 98.71 | <10 | <2 | 619 | 597 | 96.45 | 22 | 3.55 | 619 |
|  |  | 4-7 days | 1385 | 99.07 | 13 | 0.93 | 1398 | 1345 | 96.21 | 53 | 3.79 | 1398 |
|  |  | 8-14 days | 493 | 99 | <10 | <1 | 498 | 473 | 94.98 | 25 | 5.02 | 498 |
|  |  | 15-27 days | 105 | 98.13 | <10 | <10 | 107 | 101 | 94.39 | <10 | <10 | 107 |
|  |  |  |  |  |  |  |  |  |  |  |  |  |
| Elderly ≥75 years | Interval length | 0-3 days | 182 | 94.3 | 11 | 5.7 | 193 | 165 | 85.49 | 28 | 14.51 | 193 |
|  |  | 4-7 days | 417 | 94.34 | 25 | 5.66 | 442 | 380 | 85.97 | 62 | 14.03 | 442 |
|  |  | 8-14 days | 151 | 96.18 | <10 | <6 | 157 | 135 | 85.99 | 22 | 14.01 | 157 |
|  |  | 15-27 days | 55 | 100 | - | - | 55 | 49 | 89.09 | <10 | <20 | 55 |
