# Supplementary material for: Time to Surgery Following Short-Course Radiotherapy in Rectal Cancer and its Impact on Postoperative Outcomes. A Population-Based Study Across the English National Health Service, 2009–2014
Source: Clin Oncol (R Coll Radiol). 2020 Feb;32(2):e46–52. doi: 10.1016/j.clon.2019.08.008 (PMC6966322; doi:10.1016/j.clon.2019.08.008)
Supplement: Multimedia component 6 [file mmc6.docx]

**Supplementary Table S5**

Associations between interval length and thirty day mortality and one-year survival, independently for not elderly and elderly patients. Adjusted models are adjusted for stage, co-morbidity, sex and IMD.

| Not elderly patients (<75 years of age) | | | | | | | | | | | Elderly patients (≥75 years of age) | | | | | | | | | |
| --- | --- | --- | --- | --- | --- | --- | --- | --- | --- | --- | --- | --- | --- | --- | --- | --- | --- | --- | --- | --- |
|  | 30 day mortality - logistic regression | | | | | | 1 year survival – Cox Proportional Hazards | | | | 30 day mortality - logistic regression | | | | | | 1 year survival – Cox Proportional Hazards | | | |
|  | Unadjusted | | | | | |  | | | |  | | | | | |  | | | |
|  |  | OR | CI - lower | CI - higher | | P | HR | CI - lower | CI - higher | P (z)  (1 year) | OR | CI - lower | CI - higher | | P | | HR | CI - lower | CI - higher | P (z)  (1 year) |
| Interval length | 0-3 days | *Reference* | |  | |  | *Reference.* | |  |  | *Reference* | |  | |  | | *Reference* | |  |  |
|  | 4-6 days | 0.72 | 0.30 | 1.74 | | 0.46 | 1.06 | 0.65 | 1.75 | 0.81 | 0.99 | 0.48 | 2.06 | | 0.98 | | 0.96 | 0.61 | 1.50 | 0.85 |
|  | 7-14 days | 0.77 | 0.25 | 2.38 | | 0.66 | 1.42 | 0.80 | 2.51 | 0.23 | 0.66 | 0.24 | 1.82 | | 0.42 | | 0.96 | 0.55 | 1.68 | 0.90 |
|  | 15-27 days | *^*^* |  |  | |  | 1.60 | 0.65 | 3.94 | 0.31 | *^*^* |  |  | | |  | 0.72 | 0.30 | 1.75 | 0.47 |
|  | *Baseline* | 0.01 | 0.01 | 0.03 | | <0.01 |  |  |  |  | 0.06 | 0.03 | 0.11 | | <0.01 | |  |  |  |  |
|  | Adjusted | | | | | |  | | | |  | | | | | |  | | | |
|  |  | OR | CI - lower | CI - higher | | P | HR | CI - lower | CI - higher | P (z) (1 year) | OR | CI - lower | CI - higher | | P | | HR | CI - lower | CI - higher | P (z)  (1 year) |
| Interval length | 0-3 days | *Reference* | |  | |  | *Reference* | |  |  | *Reference* | |  | |  | | *Reference* | |  |  |
|  | 4-6 days | 0.70 | 0.29 | 1.72 | | 0.44 | 1.05 | 0.64 | 1.73 | 0.84 | 0.86 | 0.41 | 1.82 | | 0.70 | | 0.88 | 0.56 | 1.38 | 0.59 |
|  | 7-14 days | 0.77 | 0.25 | 2.40 | | 0.65 | 1.32 | 0.74 | 2.35 | 0.34 | 0.57 | 0.20 | 1.60 | | 0.28 | | 0.93 | 0.53 | 1.62 | 0.79 |
|  | 15-27 days | *^*^* |  |  |  | | 1.48 | 0.59 | 3.67 | 0.40 | *^*^* |  |  |  | | | 0.56 | 0.23 | 1.38 | 0.21 |
|  | Baseline | 0.00 | 0.00 | 0.03 | | <0.01 |  |  |  |  | 0.04 | 0.01 | 0.14 | | <0.01 | |  |  |  |  |

^*^ Not included as no deaths by 30 days in the elderly group.
